# The Effect of Active Recovery on Power Performance During the Bench Press Exercise

**DOI:** 10.2478/hukin-2014-0018

**Published:** 2014-04-09

**Authors:** Felipe A. S. Lopes, Valéria L. G. Panissa, Ursula F. Julio, Elton M. Menegon, Emerson Franchini

**Affiliations:** 1 Center for Biological and Health Science – University Presbyterian Mackenzie, São Paulo, Brazil.; 2 Department of Sport, School of Physical Education and Sport, University of São Paulo, São Paulo, Brazil.

**Keywords:** recovery type, blood lactate concentration, bench press, power

## Abstract

The objective of this study was to verify the effect of active and passive recovery on blood lactate concentration and power performance. Twelve male subjects were submitted to a maximal strength test in the the bench press, a maximal aerobic test in the bench step, and to four sets of bench press exercise performed as fast and as long as possible, using 80% of maximal strength when active or passive recovery was performed. The maximum number of repetitions, mean and peak power in eccentric and concentric phases were computed and blood lactate concentration was measured. Comparisons for the variables were made using a two-way variance analysis (recovery type and set numer) with repeated measures in the second factor. When significant differences were detected (p < 0.05), a Tukey post-hoc test was used. There was a main effect of set number on maximum number of repetitions (p < 0.05) (1 > 2, 3, and 4; 2 > 3 and 4; 3 > 4). Mean and peak power in both eccentric and concentric phases also differed across sets (1 > 2, 3, and 4; 2 > 4). There was also a main effect for the recovery type, with lower values (p < 0.05) observed for the active recovery compared to the passive one. It can be concluded that active recovery resulted in lower lactate concentration, but did not improve power performance in the bench press exercise.

## Introduction

Improvement of muscle strength is an important goal for athletes (Tan, 2002) and physically active individuals (Garber et al., 2011), and interest in evaluating training methods to maximize strength gains has been increasing. A single session of strength training is composed of periods of work (sets) and rest (recovery), and although several studies have attempted to determine the best recovery interval between sets (De Salles et al., 2009), intensity, and total number of sets (Peterson et al., 2004), few investigations have focused on the type of recovery between sets (Corder et al., 2000; Hannie et al., 1995; Mohamad et al., 2012).

Since the mechanical stimulus is an important factor in strength development (Mohamad et al., 2012), increasing the number of repetitions and the total volume may be important factors in optimizing the training load. There is evidence that active recovery can be a good strategy to improve performance in a single session of strength training, and one factor that could explain this improvement is the increased lactate removal promoted by active recovery compared to that of passive recovery (Corder et al., 2000).

Several researchers have suggested that the excessive accumulation of blood lactate concentration ([LA]) hinders physical performance because an increase in hydrogen ions (H^+^) is thought to delay glycolysis activation by inhibiting enzyme activity or interfering with the muscle contraction process, resulting in fatigue and the consequent interruption of the activity (Fitts, 1994). However, a definitive relationship between LA concentration or H^+^ ions and a decrease in performance has not been established directly (Monedero and Donne, 2000) because other studies have shown no significant difference in performance when the concentrations of blood lactate and H^+^ ions are markedly elevated and when they are not (Franchini et al., 2003; Franchini et al., 2009; Mohamad et al., 2012; Weltman and Regan, 1983; Weltman et al., 1979).

Based on the assumption that blood lactate and H^+^ ions are associated with a decrease in performance, a number of studies have explored the recovery strategies used to decrease lactate concentration, including active recovery (Corder et al., 2000; Gupta et al., 1996), oxygen inhalation (Weltman et al., 1979), compression garment with active recovery (AR) (Lovell et al., 2011), massage (Gupta et al., 1996; Monedero and Donne, 2000; Zelikowski et al., 1993), and combined (AR and massage) (Monedero and Donne, 2000). Although it is clear that active recovery increases lactate removal more than does passive recovery (Gupta et al., 1996; Taoutaou et al., 1996; Weltman et al., 1977), there are doubts about the influence these types of recovery have on subsequent performance, especially because these studies have methodological differences in relation to the task that is used as the performance criterion.

Two studies focusing specifically on strength exercise showed increased performance (maximum number of repetitions) when active recovery was used compared with passive recovery, and one of them also showed a concomitant decrease in LA concentration (Corder et al., 2000), while the other did not (Hannie et al., 1995). Conversely, Mohamad et al. (2012) found that inter-set active recovery did not have significant influence on LA concentration and strength performance (measured as force and power).

However, the exercise that was used for active recovery (cycling) in the aforementioned studies is known to result in a lower venous return compared to exercises performed in a standing position (e.g., running, stepping) (Galy et al., 2003). Thus, standing exercises should be used to optimize blood flow during active recovery. Moreover, only one study has tested power performance after this type of intervention (Mohamad et al., 2012), and it focused on the lower body. Thus, the objective of this study was to determine the effects of active and passive recovery on LA concentration and power performance in the bench press exercise using the bench step as the equipment for recovery.

## Material and Methods

### Participants

Twelve recreationally trained males (age: 22.3 ± 3.2 years; body height: 179.1 ± 7.8 cm; body mass: 79.0 ± 11.0 kg) volunteered to participate in this study. All of the subjects were nonsmokers and were considered injury-free. They had been active in recreational sports (soccer, volleyball, and basketball) for at least 1 year, but they had no formal strength or endurance training experience. Before the beginning of the study, the subjects received a verbal explanation of all experimental procedures and risks and signed an informed consent form. The study was approved by the Mackenzie Presbyterian University ethics committee.

### Procedures

To investigate the effectiveness of active recovery on power during the bench press exercise, twelve recreationally trained males were recruited to perform four sessions with at least 72 hours of rest between each session. The participants completed the following tests over the course of three weeks: (a) a maximum strength test (1RM), (b) a progressive incremental test on a bench, and (c) 2 experimental sessions performed randomly on different days, with an active (bench step) or passive (seated) recovery undertaken between sets. The variables analyzed were LA concentration, the number of repetitions, as well as the mean and peak power generated in each set. Because previous findings suggest that active recovery is associated with greater lactate removal between sets (Gupta et al., 1996; Taoutaou et al., 1996), the bench step exercise was used to optimize the recovery. The bench step may be performed under different settings regardless of recovery time and is an exercise performed with large muscle groups, which should facilitate LA removal.

#### Maximum strength test

In the first session, the maximum load at which the subject was able to perform only one movement (1RM) for the bench press exercise (maximum dynamic strength) was determined by following the recommendations of the American Society of Exercise Physiology (Brown and Weir, 2003). The subjects began the test with a general warm-up consisting of cycling (70 rpm at 50 W) for 5 min. They subsequently performed a set of eight repetitions at an intensity of 50% of the estimated 1RM load, followed by another set of three repetitions at 70% of the estimated 1RM load; the interval between the warm-up sets and first 1RM trial was two minutes. The attempt to establish the 1RM was carried out by lifting progressively heavier loads. The first attempt started with the estimated 1RM load, and the load was increased by 5% for each subsequent lift until voluntary exhaustion, considered to be the point at which the subject was unable to complete a single repetition through the full range of motion. The rest interval was three to five minutes, and the number of attempts did not exceed five. During the 1RM test, the standard movement was established as the hand grip on the bar and the complete extension of the arms.

### Progressive Incremental test

The subjects performed an incremental step bench test to volitional exhaustion to determine their lactate threshold and the onset of blood lactate accumulation (OBLA). The initial load was set at 15 W. Each stage lasted 5 minutes and was increased by 15 W per stage until the subject could no longer continue. To calculate the power of the bench step, the following procedures were adopted: determination of body mass, step height and frequency increases [Power = strength (N) × speed (m/s)]. Thus, the speed was adjusted individually in each stage to generate the necessary intensity (15 W).

Prior to the first stage and at the end of each stage, LA concentration was determined with a portable lactate analyzer (Accusport, Roche, Brazil). The lactate threshold was considered the last period in which the LA concentration did not differ by more than 1 mmol·l^−1^ from the rest value. The OBLA was calculated assuming a fixed concentration of 4 mmol·l^−1^ (Svedahl and McIntosh, 2003). The test was terminated when two consecutive LA measurements were higher than 4 mmol·l^−1^.

### Experimental sessions

*S*ubjects were randomly allocated and performed the following workout: a general warm-up on the bench step for five minutes at OBLA intensity, followed by a 1-min rest interval, and then a specific warm-up on the bench press consisting of 12 repetitions at 40% of their 1RM. After a two-min rest period, the subjects performed four sets as fast as possible with a load that was 80% of their 1RM. The rest interval between consecutive sets was 3 min. During the recovery period between sets, the subjects performed active recovery (intensity corresponding to lactate threshold) or passive recovery (PR) (remaining seated). Blood samples were collected from the ear lobe and were immediately analyzed using a portable lactate analyzer (Accusport, Roche, Brazil) to determine LA concentration. LA concentration was measured at rest, after a warm-up, before set 1, and after sets 1, 2, 3, and 4 (two minutes after the final set of the bench press).

The number of repetitions correctly performed was computed, and the mean and peak power in the concentric and eccentric phase were calculated. The peak and mean power during each repetition were measured using the Encoder Linear Peak Power 4.0 (CEFISE, Campinas, Brazil). This equipment uses a cable positioned on the bar through which displacement is registered electronically, converted digitally, and transferred to the computer. The displacement is recorded in millimeters, and a chronometer registers the time in microseconds (1 × 10^−6^ seconds). The software analyzes the load, time, and displacement information and calculates the velocity, acceleration, and power. These calculations are conducted in both concentric and eccentric phases. Before the measurements, the equipment is calibrated using a known distance (1 m), which is used as a reference for all other displacements. In high-speed resistance exercise, this equipment has a reliability of 0.95 using the intraclass coefficient correlation.

### Statistical Analysis

The data were analyzed using the Statistical Package for Social Sciences 18.0 (SPSS Inc., Chicago, United States of American). The descriptive analyses consisted of the mean and standard deviation. For all of the measured variables, the sphericity estimated was verified according to the Mauchly’s W test, and the Greenhouse–Geisser correction was used when necessary. The data normality was verified using the Shapiro-Wilk test.

The comparison of LA values at rest, post warm-up and pre set 1 in the different types of recovery was conducted through a two-way analysis of variance (recovery type and moment), with repeated measurements in the second factor. Moreover, the comparison of the total maximum number of repetitions, mean and peak power in concentric and eccentric phases and LA concentration in the different conditions was conducted through a two-way analysis of variance (recovery type and set number) with repeated measurements in the second factor. When a significant difference or interaction was observed, the Tukey’s post hoc test was conducted. The effect size (eta-squared; η^2^) of each test was calculated for all analyses. Statistical significance was set at *p* < 0.05.

## Results

Table 1 presents the maximum number of repetitions performed using the different types of recovery for each set of the bench press exercise.

There was a significant main effect for the sets in the maximum number of repetitions (F_3,66_ = 99.54; *p* < 0.001; η^2^ = 0.819), with higher values observed during set 1 compared to sets 2, 3, and 4 (*p* < 0.001 for all comparisons), set 2 compared to sets 3 and 4 (*p* < 0.001 for all comparisons), and set 3 compared to set 4 (*p* = 0.006). Recovery type had no effect (*p* > 0.05).

Table 2 presents the main results of mean power and peak power during the concentric and eccentric phases in the different recovery types for each set of the bench press exercise.

There was a significant main effect observed for the sets in peak power (F_3,66_ = 46.20; p < 0.001; η^2^ = 0.677) and mean power (F_3,66_ = 26.27; p < 0.001; η^2^ = 0.540) during the concentric phase, with higher values observed during set 1 compared to sets 2, 3, and 4 (*p* < 0.001 for all comparisons), and set 2 compared to set 4 (*p* < 0.001). The recovery type had no effect (*p* > 0.05).

There was a significant main effect for sets in peak power (F _3,66_ = 17.60; p < 0.001; η^2^ = 0.445) and mean power (F _3,66_ = 16.23; p < 0.001; η^2^ = 0.425) during the eccentric phase, with higher values observed during set 1 compared to sets 2, 3, and 4 (*p* < 0.001 for all comparisons), and set 2 compared to set 4 (*p* < 0.001). There was no main effect of recovery type (*p* > 0.05).

The warm-up and pre-exercise movements showed an effect on LA in both groups (F_2,44_ = 83.07; p < 0.001; η^2^ = 0.791), with lower values observed at rest (2.1 ± 0.3 mmol·L^−1^) compared to post warm-up (3.2 ± 0.8 mmol·L^−1^) and pre set 1 (3.6 ± 0.6 mmol·L^−1^) (*p* < 0.001 for both comparisons), and post warm-up compared with pre set 1 (*p* = 0.001).

Table 3 presents LA concentration after each set of bench press exercise in the different recovery types.

There was a significant main effect for sets concerning LA concentration (F_3,66_ = 7.2; p < 0.001; η^2^ = 0.248), with lower values observed post set 1 compared with post set 2 (*p* = 0.006), post set 3 and post set 4 (*p* = 0.001); post set 2 [La] was lower compared to post set 4 (*p* = 0.046). There was also a significant main effect observed for the recovery type (F_1,22_ = 26.41; p < 0.001; η^2^ = 0.546 ), with higher values registered during passive recovery compared to active recovery (*p* < 0.001).

## Discussion

The main finding of this study was that although AR more effectively removed LA than PR, no performance difference was observed during the four sets of the bench press exercise. Several studies have analyzed the effectiveness of AR and PR, but most of them focused on endurance and intermittent exercises (Franchini et al., 2003; Gupta et al., 1996; Taoutaou et al., 1996; Weltman et al., 1979), and only a few studies focused specifically on strength exercises (Corder et al., 2000; Hannie et al., 1995; Mohamad et al., 2012). Among these studies, Hannie et al. (1995) and Corder et al. (2000) observed improved performance on subsequent sets after AR, whereas Mohamad et al. (2012) did not observe significant differences.

Hannie et al. (1995) conducted a study with 15 untrained subjects who performed 4 sets of the bench press exercise (at 65% of their 1RM once every 5 s) interspersed with AR or PR. The AR consisted of 1 minute on a bicycle ergometer at 45% VO_2_ max, while during PR, the subjects remained inactive between sets. The isometric bench press force was assessed prior to and after each set and after recovery. The main finding was improved performance when AR was adopted. The PR demonstrated a total decrease of 27 repetitions, while in the AR group, a decrease of only 23 repetitions was noted. However, no differences were found in isometric force between conditions, and moreover, LA concentrations were not different between the AR and PR groups and increased along sets, which may have occurred because the very short rest period between sets was not sufficient for recovery.

Using 3 different strategies of recovery (cycling at 25% and 50% of the onset of blood lactate (OBLA), and passive sitting), Corder et al. (2000) conducted a study with 15 resistance-trained males who performed 6 sets of 10 repetitions at 85% of 10RM in the parallel squat with 4 minutes of rest between bouts. To assess the efficiency of each intervention, a maximal repetition performance test (MRP) using 65% of their 10RM was undertaken following the last recovery period of each parallel squat workout, that is, the subjects performed a maximum number of repetitions with a preset load. The authors observed a greater lactate removal using the AR at 25% OBLA compared with 50% OBLA and passive rest. Importantly, this study found a negative correlation between elevated blood lactate concentration and results of the MRP test (r = −0.70 at 25% of the OBLA; r = −0.65 at 50% of the OBLA; r = −0.72, p < 0.01 at passive recovery), indicating that the higher lactate concentration was related to decreased performance.

Mohamad et al. (2012) studied recreationally trained subjects performing the squat exercise with two load schemes (3 sets of 12 repetitions at 70% of 1RM and 6 sets of 12 repetitions at 35% of 1RM). Both AR and PR were assayed with each loading scheme. The AR was described as self-selected resistance with velocity between 50–70% rpm and a heart rate of 50–60% of the maximum heart rate for 90 s (performed between sets). They did observe significant differences in the average force, peak force, average power, peak power, total work, total impulse, and lactate removal with the different types of recovery for both load schemes. However, the limitations of this study can call into question the impact of the results. The load used for the active recovery may not stimulate the necessary intensity for an enhancement of performance and a lower lactate concentration, and an individual aerobic threshold would have been more appropriate for stimulating the sought-after adaptations, and this study did not use a protocol with a maximum number of repetitions to assess performance.

As detailed above, the findings of the present study differ from those of the studies cited above, which may be explained by several factors, including the methodological design, recovery time between work bouts, characteristics of subjects, and intensity of the exercise utilized. The other factor that confounds comparisons among studies is the type of exercises and muscle groups analyzed. The present study analyzed performance in the bench press, using the bench step as a recovery exercise, while Hannie et al. (1995) also analyzed performance in the bench press but used the cycle ergometer as the recovery exercise, and finally, Corder et al. (2000) analyzed performance in the squat, using the cycle ergometer as the recovery exercise.

The present study utilized a type of recovery focusing on a different muscle group than that utilized in the exercise, which may optimize a lower blood lactate concentration, according to Thiriet et al. (1993). However, further studies using different types of recovery exercises are needed to confirm this hypothesis and determine whether active recovery using a different muscle group can also improve performance.

Despite the enhanced lactate removal rates after AR, the data in the present study are in agreement with several authors studying the use of AR, where the overall lactate concentration using AR was greater than that observed with PR (Corder et al., 2000; Gupta et al., 1996; Taoutaou et al., 1996). However, the lower lactate concentration after the AR did not cause a performance improvement in the present study. Hannie et al. (1995) also did not find any relationship between lactate removal rates and performance improvement. These data suggest that the relationship between lactate removal and performance can be occasional, at least in situations involving active recovery and strength exercises.

The conflicting observations regarding performance and different concentrations of blood lactate and H^+^ ions seem to be associated with issues such as recovery time between work bouts over 15 minutes (Bond et al., 1991; Franchini et al., 2009; Watts, et al., 2000; Weltman et al., 1979; Weltman and Regan, 1983) and lower than 6 minutes (Bogdanis et al., 1996; Connolly et al., 2003; Corder et al., 2000; Mika et al., 2007), characteristics of the subjects (trained (Corder et al., 2000), untrained (Hannie et al., 1995) and recreationally trained (Mohamad et al., 2012)), task characteristics (intermittent anaerobic task) (Franchini et al., 2003; Franchini et al., 2009), weight training (Corder et al., 2000; Hannie et al., 1995; Mohamad et al., 2012) and the specificity of the task in relation to the training of the subjects, such as swimmers (Siebers and Mcmurray, 1981), judo athletes (Franchini et al., 2003), endurance-trained or sprint-trained athletes (Taoutaou et al., 1996).

After a rest period over 15 minutes, performance has been shown to be similar to the control condition, independent of recovery strategy (Bond et al., 1991; Franchini et al., 2009; Watts et al., 2000; Weltman et al., 1979; Weltman and Regan, 1983), indicating that the time of the rest period, in addition to blood lactate and the type of recovery, may be one of the keys to performance recovery. Moreover, a short rest period (< 6 minutes), as used in most studies, improved the performance after active recovery compared to passive recovery (Bogdanis et al., 1996; Connolly et al., 2003; Corder et al., 2000; Mika et al., 2007), despite the blood lactate concentrations not necessarily being different between the two recovery strategies (Bogdanis et al., 1996; Signorile et al., 1993) and the time not being long enough to recover after the previous performance (Bogdanis et al., 1996), suggesting that factors other than blood lactate concentration may contribute to the benefits of active recovery in situations with short duration.

Active recovery using the bench step was able to promote greater blood lactate removal compared to passive recovery, but no effect on strength performance in the bench press was observed. Given that previous studies have shown improved strength performance after active recovery, recovery strategies manipulating additional variables (such as the ergometer type, intensity and time of recovery, and the combination of muscles engaged) merit further research.

## Figures and Tables

**Table 1:** Maximum number of repetitions performed during four sets of the bench press exercise using two different types of recovery

	1	2^a^	3^ab^	4^abc^
PR	10 ± 2	7 ± 2	5 ± 2	4 ± 2
AR	10 ± 2	6 ± 2	4 ± 2	4 ± 1

PR: passive recovery; AR: active recovery;

a: significant difference from set 1 (p < 0.05);

b: different from set 2 (p < 0.05);

c: different from set 3 (p < 0.05).

**Table 2:** Mean power (MP) and peak power (PP) during concentric and eccentric phase performed during four sets of the bench press using two different types of recovery

		1	2^a^	3^a^	4^ab^
Concentric phase					

PR	PP (W)	376.7 ± 110.6	297.9 ± 77.1	248.8 ± 94.2	216.7 ± 74.9
	MP (W)	259.1 ± 58.6	211.2 ± 53.7	194.9 ± 73.7	178.2 ± 60.5

AR	PP (W)	336.1 ± 60.7	280.2 ± 66.9	247.3 ± 54.5	216.6 ± 56.8
	MP (W)	237.8 ± 41.2	206.3 ± 45.8	199.7 ± 39.9	172.0 ± 37.5

Eccentric phase					

PR	PP (W)	−437.3 ± 101.3	−380.2 ± 100.1	−344.7 ± 96.8	−335.6 ± 119.6
	MP (W)	−351.8 ± 90.3	−306.3 ± 96.7	−282.1 ± 103.1	−271.7 ± 116.5

AR	PP (W)	−431.6 ± 90.4	−401.2 ± 93.3	−386.9 ± 101.7	−345.6 ± 67.0
	MP (W)	−344.9 ± 69.1	−325.0 ± 73.7	−322.6 ± 84.8	−272.6 ± 52.6

PR: passive recovery passive; AR: active recovery;

a: different from set 1 for PP and MP (p < 0.05);

b: different from set 2 for PP and MP (p < 0.05).

**Table 3:** Blood lactate concentration after each set of the bench press exercise following different recovery conditions

	Passive^c^	Active
Post set 1 (mmol·L^−1^)	5.5 ± 0.6	4.7 ± 0.8
Post set 2 (mmol·L^−1^)^a^	6.2 ± 0.9	4.9 ± 0.6
Post set 3 (mmol·L^−1^)^a^	6.6 ± 1.1	5.0 ± 0.6
Post set 4 (mmol·L^−1^)^ab^	6.8 ± 1.4	5.4 ± 0.7

a: different from set 1 (p < 0.05);

b: different from set 2;

c: different from active recovery (p < 0.05).
